# Online detection of compensatory strategies in human movement with supervised classification: a pilot study

**DOI:** 10.3389/fnbot.2023.1155826

**Published:** 2023-07-14

**Authors:** Neha Das, Satoshi Endo, Sabrina Patel, Carmen Krewer, Sandra Hirche

**Affiliations:** ^1^Information-Oriented Control, TUM School of Computation, Information and Technology, Technical University of Munich, Munich, Germany; ^2^Human Movement Science, Department of Sports and Health Sciences, Technical University of Munich, Munich, Germany; ^3^Department of Neurology, Research Group, Schoen Clinic Bad Aibling, Bad Aibling, Germany

**Keywords:** compensation detection, stroke rehabilitation, machine learning, bio-mechanical features, supervised classification

## Abstract

**Introduction:**

Stroke survivors often compensate for the loss of motor function in their distal joints by altered use of more proximal joints and body segments. Since this can be detrimental to the rehabilitation process in the long-term, it is imperative that such movements are indicated to the patients and their caregiver. This is a difficult task since compensation strategies are varied and multi-faceted. Recent works that have focused on supervised machine learning methods for compensation detection often require a large training dataset of motions with compensation location annotations for each time-step of the recorded motion. In contrast, this study proposed a novel approach that learned a linear classifier from energy-based features to discriminate between healthy and compensatory movements and identify the compensating joints without the need for dense and explicit annotations.

**Methods:**

Six healthy physiotherapists performed five different tasks using healthy movements and acted compensations. The resulting motion capture data was transformed into joint kinematic and dynamic trajectories. Inspired by works in bio-mechanics, energy-based features were extracted from this dataset. Support vector machine (SVM) and logistic regression (LR) algorithms were then applied for detection of compensatory movements. For compensating joint identification, an additional condition enforcing the independence of the feature calculation for each observable degree of freedom was imposed.

**Results:**

Using leave-one-out cross validation, low values of mean brier score (<0.15), mis-classification rate (<0.2) and false discovery rate (<0.2) were obtained for both SVM and LR classifiers. These methods were found to outperform deep learning classifiers that did not use energy-based features. Additionally, online classification performance by our methods were also shown to outperform deep learning baselines. Furthermore, qualitative results obtained from the compensation joint identification experiment indicated that the method could successfully identify compensating joints.

**Discussion:**

Results from this study indicated that including prior bio-mechanical information in the form of energy based features can improve classification performance even when linear classifiers are used, both for offline and online classification. Furthermore, evaluation compensation joint identification algorithm indicated that it could potentially provide a straightforward and interpretable way of identifying compensating joints, as well as the degree of compensation being performed.

## 1. Introduction

Stroke is one of the leading causes for long-term disability worldwide (Murray et al., 2012) and often results in upper-extremity motor impairment in survivors (Kwakkel et al., 2003) that can severely affect their quality of life and health (Franceschini et al., 2010; Morris et al., 2013). Hence, regaining upper-limb function post-stroke is vital for patient recuperation and consequently, is a major target of rehabilitative-therapy. In particular, repetitive and task specific training of the affected limbs have been suggested to be one of the main drivers of rehabilitation (Bütefisch et al., 1995; Dickstein et al., 1997). Training has traditionally been conducted with assistance and feedback from physiotherapists in a clinical setting. However, this requires constant monitoring and guidance by the physiotherapist, a task that becomes difficult with the increasing number of patients (Pollock et al., 2000). While this can be addressed in part by recommending exercises to the patient for in-home rehabilitation at later stages of recovery (Turton and Fraser, 1990), adopting such an approach introduces novel challenges - namely, providing appropriate feedback to the patient regarding their performance.

Recently, efforts have been made to this end, as well as to alleviate the physiotherapists' workload in clinical settings by introducing automation into the rehabilitation pipeline, for instance via robot-assisted therapy (Aprile et al., 2020; Takebayashi et al., 2022) and interactive game-based therapy (Laver et al., 2017; Laffont et al., 2020). These techniques must be equipped with evaluation mechanisms that can automatically assess the quality and success of the ongoing rehabilitation exercise or task performed by patients, ideally in an online manner, in order to provide useful feedback for facilitating improvement in real-time. However, such an automatic quantification of task performance can be challenging. While the success of task or exercise completion is relatively simple to track and quantify automatically—for example by tracking the distance between the end-point of the impaired limb and the goal position, it might not provide an adequate picture of performance, especially with regards to the reappearance of premorbid motor behavior (Cirstea and Levin, 2007). This complication arises in part from the use of compensatory strategies by the patient in the post-stroke period.

Stroke patients often compensate for the impairment caused in one joint by overusing an unimpaired joint for the successful accomplishment of rehabilitation exercises or activities of daily living (Cirstea and Levin, 2000). Any redundant joint that is relatively underused for a particular motion or task can be recruited when the typically used joint is impaired to ensure successful completion of the task. The degree of compensation provided by the recruited joint can vary from mild to severe (Cirstea and Levin, 2000). It has been noted that the long-term use of compensatory strategies can interfere with rehabilitation goals (Takeuchi and Izumi, 2012). Accurate and automatic identification of compensatory strategies and deviation from healthy motion is therefore an integral part of monitoring exercise/task performance during a therapy session. Moreover, inclusion of this information has been found to be helpful by the patients (Fruchter et al., 2022).

Most of the research toward automatic compensation detection has been geared toward exploiting data-driven supervised learning methods for the task. In general, such methods rely on the availability or acquisition of a dataset of motions which are labeled by experts to be either healthy or compensatory. The acquired dataset of motions is used to train a machine learning model, which can be used afterwards for classifying observed motions at test time. Previous works have explored a variety of machine-learning architectures and models for this task, ranging from decision-trees (Sellmann et al., 2022) and non-parametric methods, such as k-Nearest Neighbor classification (Cai et al., 2019) and Support Vector Machine classifiers (Taati et al., 2012; Zhi et al., 2017) to parametric deep learning methods, such as a Multi-layer Perceptron (MLP) (Lin et al., 2021) or recurrent neural networks with Long Short-Term Memory (LSTM networks) (Zhi et al., 2017). A wide range of measurements including kinematics (Taati et al., 2012; Zhi et al., 2017; Sellmann et al., 2022), applied forces (Cai et al., 2019), and muscle activity (Ma et al., 2019) have been used as an input for these data -driven classifiers.

However, existing learning-based solutions fall short of fully addressing one or more of several common challenges posed by the task of automatic compensation detection. One major challenge is the identification of compensating joints. By leveraging multi-class classification techniques, many approachesare able to detect three common types of compensations in reaching motion—namely torso lean-forward, torso rotation and scapular elevation (Zhi et al., 2017; Cai et al., 2019; Ma et al., 2019). By design, such a classification method is geared toward detecting only one type of compensation per input motion segment, which can be a shortcoming when the motion segment contains multiple compensation strategies. Kashi et al. (2020) uses multi-label classification to mitigate this issue. However, like the preceding works, this approach relies on explicit annotations of compensation locations. This requirement can pose some limitations on the applicability of the compensation detection mechanism since providing such a detailed descriptor of the compensation strategy can be cumbersome and is subject to labeling error by the expert annotator Hickey et al. (2007). The latter can especially occur when indicators of compensatory movements are subtle and beyond the visual capabilities of physiotherapists (Abbott et al., 2022). This indicates that it is hard to find an objective measure for compensation magnitude and affected location, especially via supervised classification methods that rely on detailed annotations from experts.

Another challenge is discriminating between healthy and compensatory motions in real-time. This is particularly desirable since it can allow for the correction of a compensatory motion as it is being performed either by the means of direct feedback to the patient or through other methods such as alerting the responsible physiotherapist in case of in-clinic rehabilitation. The classification output could also be used by a robotic system to promote more desirable kinematics by means of a force feedback. However, many of the methods discussed above either train their model with pre-segmented motions and assume access to similarly segmented data during test-time (Kashi et al., 2020) or use a sliding window of fixed size for online classification (Zhi et al., 2017; Cai et al., 2019; Ma et al., 2019) which may not be able to capture long-range temporal correlations.

Lastly, most existing works for compensation detection leverage datasets that are quite small in size due to the difficulty of collecting data on a large scale from patients. For example, many works (Zhi et al., 2017; Uy and Abu, 2020; Khoramdel et al., 2021) learn from the Toronto Rehab Stroke Pose Dataset (Dolatabadi et al., 2017) that collects kinematic data from a cohort of 9 stroke survivors and 10 healthy patients, Cai et al. (2019) perform their analysis on data from 8 stroke survivors, and Lin et al. (2021) uses a dataset of motions from 10 stroke survivors. Yet, many works use deep neural networks for learning a a model for compensation detection (Zhi et al., 2017; Khoramdel et al., 2021; Lin et al., 2021). This can be counter-productive since deep learning-based architectures typically have a large number of parameters that often outnumber the small training dataset making the model susceptible to overfitting (Bishop and Nasrabadi, 2006) and reduced generalizability. Furthermore, owing to the large parameter size, these models often take longer to train. Thus, there is a growing need for data-driven methods for compensation detection that can learn from small datasets with non-explicit labels in order to decrease the reliance on manual annotation and can be applied online for obtaining predictions in real-time from streaming data.

In this work, we take a step toward closing these gaps by proposing a novel approach that learns a linear classification model that can not only discriminate between compensatory motions and healthy ones, but also identify compensating upper-body joints without requiring explicit labels in the training data. To learn an accurate classifier, selection of appropriate features is of utmost importance. In this regard, we take inspiration from bio-mechanical literature pertaining to natural motion generation and design energy-based features that can be used to learn a classifier. These features include joint jerk, power, torque rate and effort, and they are often used as proxies for metabolic energy expenditure which biomechanical models optimizes for producing natural movements (Gauthier et al., 2010; Huang et al., 2012). Thus, by including them as features, we propose that they can inform the classifier regarding the degree of atypicality of the motion. We calculate these features independently for each observed degree of freedom (DoF). This allows us to identify compensating joints in a given motion by exploiting the product of its corresponding features and the weights of the learned classifier.

We verify our approach using leave-one-out cross validation on a dataset of healthy and acted compensatory motions by qualified physiotherapists. The present study reports quantitative results that demonstrate the efficacy of our approach toward identification of compensatory motion and the degree to which the upper-body joints are attributed to such motion. Furthermore, we demonstrate that a linear classification model trained on energy-based features shows competitive performance compared to deep learning-based methods including MLP and LSTM that can automatically extract relevant features from raw data (Shaheen et al., 2016) when discriminating healthy motions from compensatory ones.

## 2. Materials and methods

### 2.1. Dataset

Six participants with no mobility impairments were recruited for this study. All participants are trained physiotherapists. Informed consent was gathered from all of the participants. Table 1 summarizes the demographic information for all the participants.

**Table 1:** Demographic information of the participants.

**Characteristics**	**Distribution (Mean ±Std.)**
Number of participants	6
Female	6
Age (years)	26 ±2.5
Weight (kg)	59 ±11.15
Height (cm)	167 ±3.93
Right-hand dominance	6

#### 2.1.1. Motion primitives

**Figure 1:**
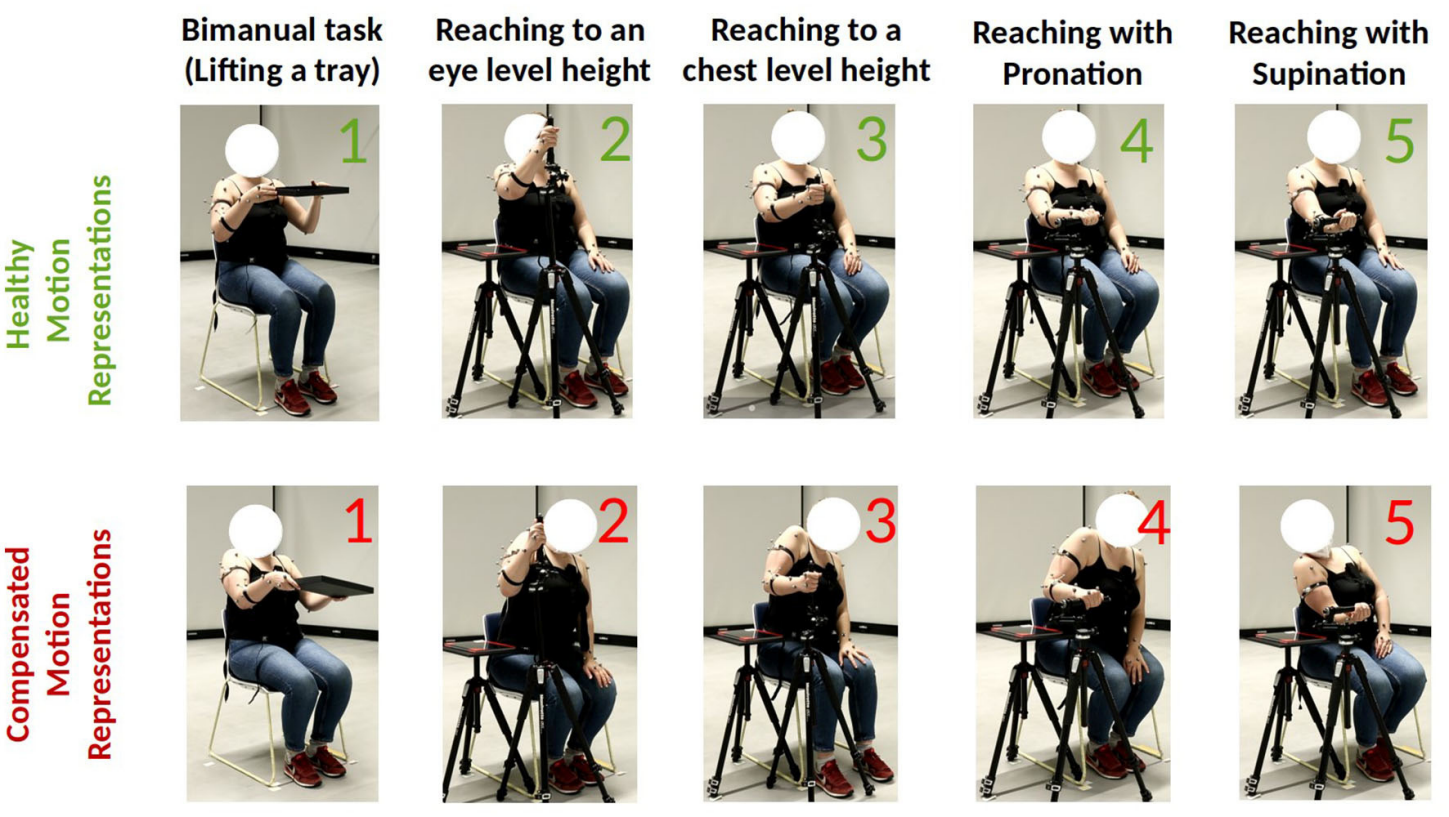
Representation of healthy and compensatory motions for the 5 primitives in our dataset.

We collected movement data for five different motion primitives, each of which corresponds to a single goal-oriented motion trajectory. These include (i) a bimanual task where the participant lifts a tray to their chest level with both arms, (ii) a unimanual task to reach and grasp an object at the eye-level, (iii) a unimanual task to reach and grasp an object at the chest-level, (iv) a unimanual task to reach and grasp an object such that it includes pronation and (v) supination. We illustrate these primitive motion patterns in Figure 1.

**Figure 2:**
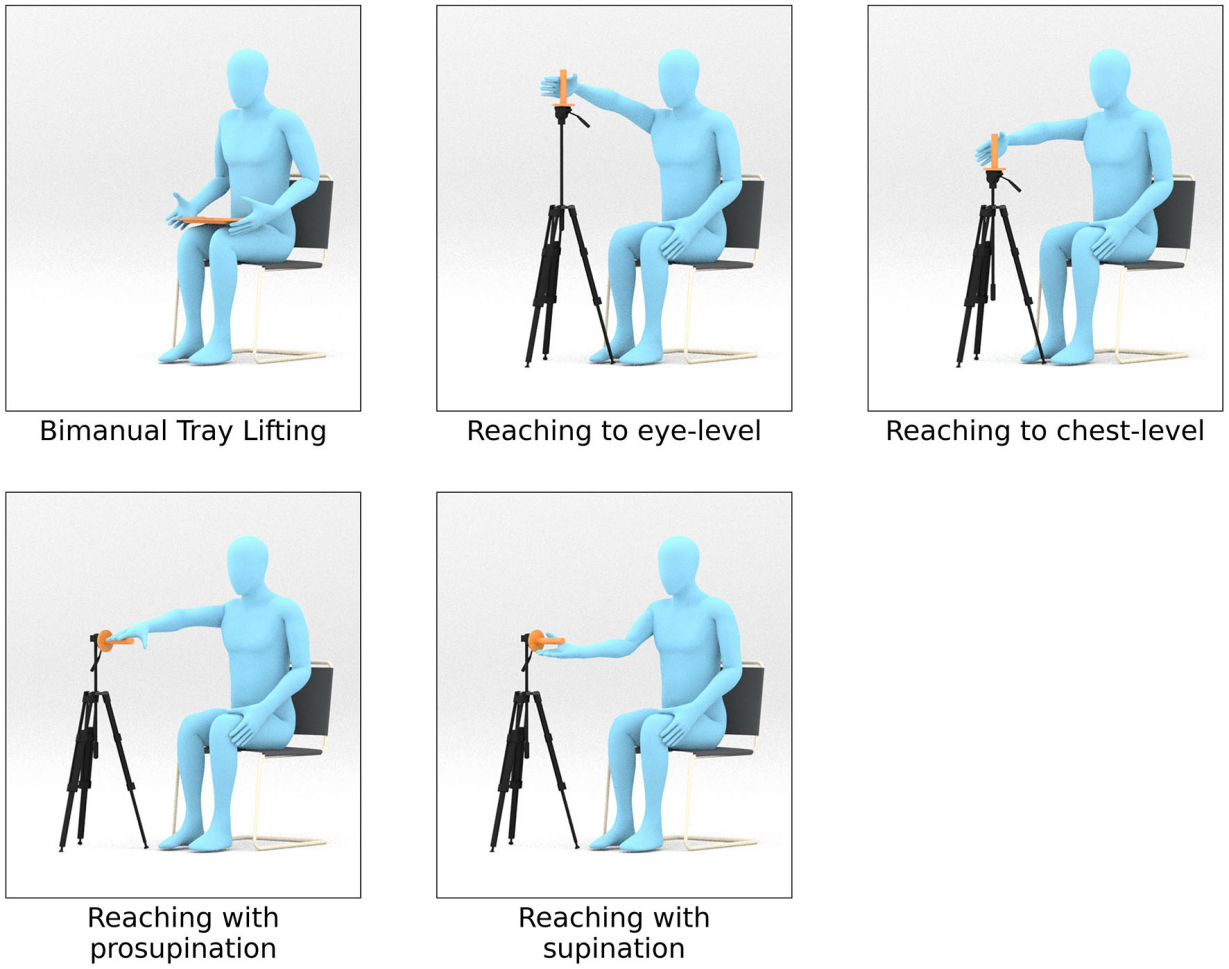
Setup for each of the 5 motion primitives. A tray is used for the bimanual lifting task. A cylindrical rod fixed at the top of a tripod is used to mark the target position for the reaching tasks. The participants were asked to grasp the cylindrical rod during the reaching tasks. For reaching to an eye-level and chest-level heights, the rod is aligned vertically. For pronation and supination tasks, the rod was rotated 90 degrees such that it was horizontal.

Repetition of such motions is often part of rehabilitation exercises (Thielman et al., 2004; Bayona et al., 2005; Rensink et al., 2009) as they comprise major motions used for performing activities of daily living. Each of these primitives display a wide range of joint activity and joint interactions both for healthy and atypical movements. The experimental setup for the bimanual tray lifting task consists of cardboard tray with dimensions 35.5 cms × 24.8 cms × 3 cms and weight 0.1 kgs. For the grasping tasks, we use a height-adjustable tripod that is mounted with a cylindrical rod of length 20 cms (the grasp object). We additionally place a side table close to the participant's dominant arm and adjust its height such that they are able to place their elbow flexed naturally at 90 degrees. We controlled for reach length among the participants by placing the tripod holding the grasp object such that it is always within reach. This is done by adjusting the position of the tripod such that its central column touches the participants' wrist when they extend their arm. At the beginning of the bimanual motion, the tray is placed on the participant's lap and grasped by its sides. The starting point for the participant's dominant arm during the reaching motions is on the side-table. Figure 2 illustrates the experimental setup for this work.

#### 2.1.2. Compensation simulation

In addition to generating natural motions corresponding to each of the 5 tasks, the participants also simulated different types of compensatory movements simulating stroke patients for each task. With regards to the latter, the participants, all of whom were physiotherapists, were instructed to enact compensations that were most commonly observed by them during their experience of interacting with stroke patients using their dominant arm. No other restrictions were placed on the type of the compensation strategies the participants could simulate. However, all of the collected motions (including the acted compensatory movement trajectories) begin with the participant sitting in a natural or “healthy” pose, with no visible joint compensations.

**Figure 3:**
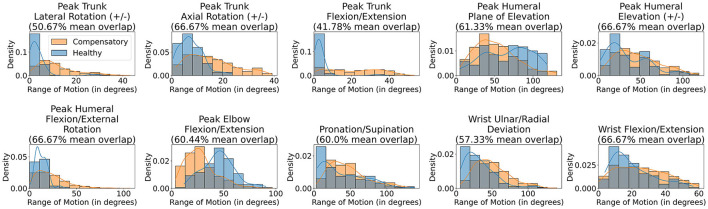
Histogram plotting the Range of Motion observed for different joints for healthy and compensatory motions.

Figure 1 illustrates the different motion primitives that comprise the dataset and compares healthy and compensated movement examples for each motion primitive. We additionally plot the distribution of range of motion (RoM) observed throughout the collected trajectories for each joint in Figure 3. RoM has been widely used by physiotherapists to assess motion health (Mortazavi and Nadian-Ghomsheh, 2019) along with other criteria. The minimum average overlap between healthy and compensatory motions was noted to be around 38 percent. This can be attributed to the fact that RoM in compensatory motion widely distributed according to the task and a person who performed it, highlighting a challenge in identifying compensatory movements using simple classification approaches, such as thresholding on RoM.

#### 2.1.3. Dataset size

We collected 5 repetitions of healthy movements and 5 repetitions of 3 acted compensatory movements for each of the 5 tasks (bimanual task, unimanual reaching to an eye level height, unimanual reaching to a chest level height, unimanual reaching with pronation, and unimanual reaching with supination) from 6 participants. This means that a total of 5 × (3+1) × 5 × 6 = 600 trajectories are collected, 100 trajectories for each individual participant.

For ensuring the variability of the healthy motions in the dataset, we calculated the width of 95% confidence intervals (C.I) for peak motions of the different joints and compared them to the 95% CI's width obtained by Gates et al. (2016) on a similar task (unimanual reaching). We found these values to be comparable to the previous work for most joints. Furthermore, for most joints, the range of peak motions in compensatory movements was found to be greater than 70% of the 95th percentile of peak motion range across all movements (where this range is given by [0, 95th percentile of peak motion]), indicating high variability of the motions in the acted compensations dataset. The 95th percentile of peak motions is calculated from McGregor et al. (1995) for trunk movements and from Gates et al. (2016) for other joints.

### 2.2. Data measurement

**Figure 4:**
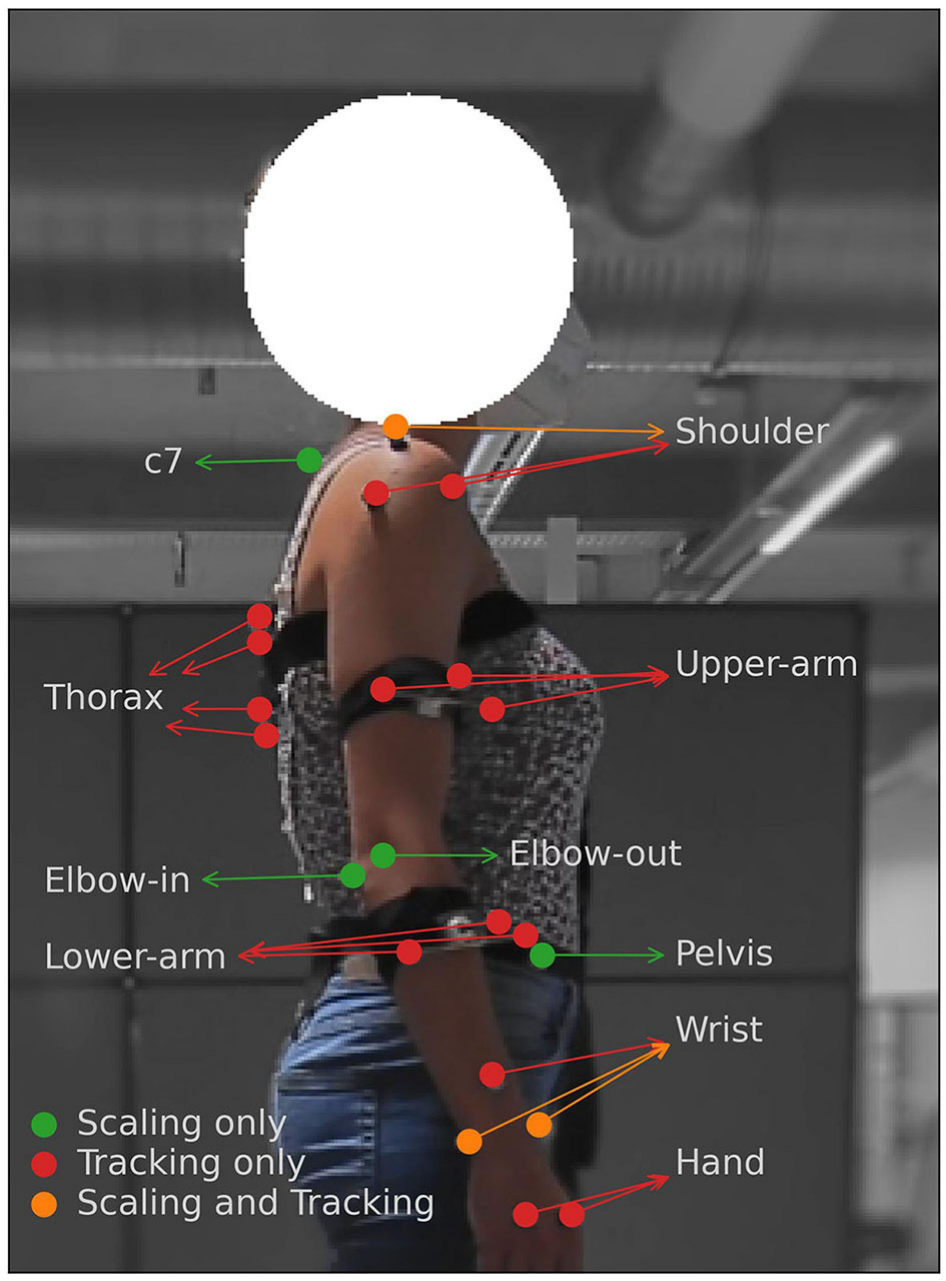
Marker placement on participants' dominant side. The placement of the upper shoulder marker, the elbow inside and outside marker, the wrist in and out markers, and the pelvis marker are mirrored on the non-dominant side of the participant, but are used only for scaling the OpenSim model.

We used Qualisys Track Manager (Senior, 2004), a marker-based motion capture system with fifteen cameras to capture the movement data of the participants. A total of 31 markers were placed on each participant. Of these, 10 markers were used purely for scaling an OpenSim (Delp et al., 2007) upper-body musculoskeletal model used for analysis (discussed below) to the participant and were removed while tracking and recording the actual motion of the participant. Figure 4 summarizes the placement and purpose of the body markers. We collected the 3D positions of the markers with respect to a common global reference frame for each of the movement trajectories generated by the 6 participants at a rate of 100 Hz.

### 2.3. Automatic motion segmentation

**Figure 5:**
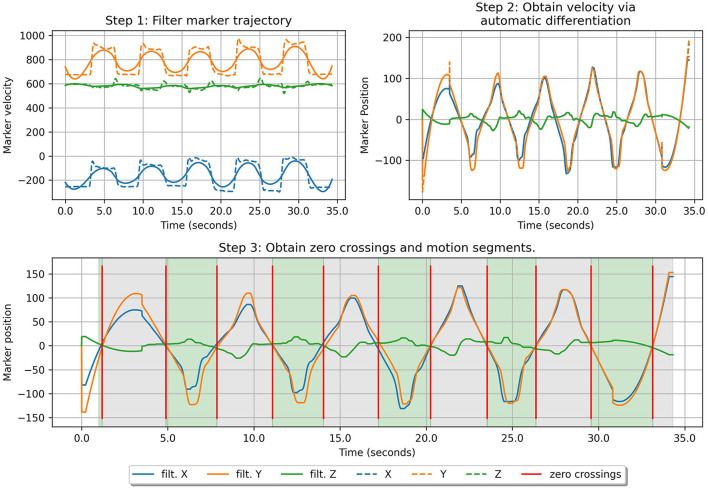
An illustration of the steps followed for automatic motion segmentation. In step 3., the green region corresponds to the execution of the motion primitives and are analyzed. The gray region corresponds to the participant moving back to their start positions and are not analyzed.

In order to streamline the process of data collection, the healthy repetitions of the distinct tasks by each physiotherapist are collected as one contiguous motion. We adopt the same approach for collecting the 3 different types of acted compensation movements from the participants. Therefore, we essentially have 4 contiguous mozion data per task per participant. From these trajectories, we automatically extract the individual motions corresponding to each of the 5 primitives with a simple approach similar to work presented in Fod et al. (2002). The following steps are performed for this purpose: (i) the marker trajectory is first smoothened with a Savitsky-Golay filter (Savitzky and Golay, 1964) of a window of length 200 milliseconds. Next, (ii) the velocity of the end-effector markers (in our case, the markers on the participants' wrists) is calculated by taking the first derivative of the marker positions. Finally, (iii) zero-crossings vector for the marker velocity trajectory are obtained. We pick the locations where velocity is 0 in all three axes. These yield the start and end locations of each individual motion. We illustrate these steps in Figure 5. We modify this approach for extracting non-segmentable motion primitives during online classification at test time as described in Section 2.9 Note that each trajectory consists of multiple repeated motions that begin from the start position, execute the motion primitive, which ends when a target configuration is reached (for example, the tray is lifted to the chest level in the bimanual task, and the cylindrical object is grasped in a particular manner for the reaching task) and return back to the start position. Since participants are not explicitly asked to follow any protocol as they move back to the start position, after executing the motion primitive, we exclude this portion of the trajectory from our analysis.

### 2.4. Data processing

We used OpenSim (Delp et al., 2007), an open source software package for modeling, simulation and analysis of human bio-mechanical systems for processing the collected data. For our analysis, we used an OpenSim biomechanical model of a human skeleton with 17 DoFs. The first three of these 17 DoFs are (i) torso flexion, which corresponds to leaning forward (ii) torso tilt, a movement that corresponds to a sideways bend of the torso, and (iii) torso rotation, which corresponds rotation of the torso about its length. The rest of the 14 DoFs correspond to the left and right arms of the participant and mirror each other. For brevity's sake, we list the DoFs for only one arm. These include (iv) elevation plane, (v) elevation angle, (vi) shoulder rotation, (vii) elbow flexion, (viii) forearm rotation, (ix) wrist flexion, and (x) wrist deviation. For detailed descriptions of these DoFs, we refer the readers to Holzbaur et al. (2005). The 3 DoFs corresponding to the torso along with the 7 arm DoFs corresponding to the dominant arm of the participants comprise the set that is used for our analysis.

**Figure 6:**
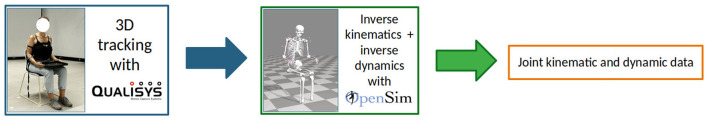
Data processing pipeline. We obtain marker-based kinematic data and process it using OpenSim to compute joint kinematic and dynamic trajectories. This is followed by extraction of energy-based features.

We will now describe our data processing pipeline (illustrated in Figure 6). First, a generic upper-body musculoskeletal model with 17 DoFs was scaled and registered to each of the 6 participants. Following this, the marker trajectories were processed using OpenSim's inverse kinematics tool to infer the corresponding joint angle trajectories of the scaled models associated with each participant. We represent the joint angle trajectories with ${\left\{q_{t}\right\}}_{t=1}^{T}$, where ***q***_*t*_ denotes the joint configuration of the musculoskeletal system at the discretized time-step *t* and *T* denotes the length of the motion trajectory. The joint configuration $q_{t}={\left\{q_{t}^{i}\right\}}_{i=1}^{N_{J}}$ is essentially a vector of relative angles corresponding to the *N*_*J*_ rotational DoFs in the musculoskeletal model of the upper body that we are using for this study. We additionally obtain trajectories of joint angle velocity ${\left\{{\overset{\cdot }{q}}_{t}\right\}}_{t=1}^{T}$ and acceleration ${\left\{{\ddot{q}}_{t}\right\}}_{t=1}^{T}$ via automatic differentiation. Finally, we used Opensim's inverse dynamics tool to derive the torques applied at each joint at each time-step (represented in this work as the joint-dynamic trajectory ${\left\{\tau_{t}\right\}}_{t=1}^{T}$) for producing the corresponding joint-kinematic trajectory ${\left\{q_{t},{\overset{\cdot }{q}}_{t},{\ddot{q}}_{t}\right\}}_{t=1}^{T}$. All collected trajectories are smoothened by using a low-pass filter with a cut off frequency of 6 Hz.

### 2.5. Feature extraction

Given the kinematic and dynamic trajectories of the motion, we obtain several metrics commonly used for analysis of human motion generation in biomechanics literature. This includes (i) angular jerk (${\ddot{q}}_{t}^{i}$), an indicator of the degree of movement smoothness in the joint space, maximization of which has been correlated to natural arm movement generation (Wada et al., 2001), (ii) power ($\left|{\overset{\cdot }{q}}_{t}^{i}\cdot \tau_{t}^{i}\right|$), (iii) effort ($\left|{\ddot{\tau }}_{t}^{i}\right|$), and (iv) torque rate ($\left|{\overset{\cdot }{\tau }}_{t}^{i}\right|$). The weighted sum of the last three metrics have been proposed by several previous works to be an indicator of metabolic cost (Zhou et al., 2017; Wong et al., 2021), minimization of which is theorized as one of the biomechanical principles for human motion (Gauthier et al., 2010; Huang et al., 2012).

We create an input feature matrix **ϕ**_*t*_ corresponding to each time-step *t* of the trajectory. This is done in two steps. First, we calculate the 4 aforementioned metrics for 10 DoFs in the musculoskeletal model separately at each time-step *t* of the movement trajectory. Next, we update each feature at time-step *t* by replacing it with cumulative averaging of all the features seen until *t* to obtain the Cumulative Averaged Energy (CAE) features. This attempts to encode temporal information in the features.

#### 2.5.1. Input normalization

**Figure 7:**
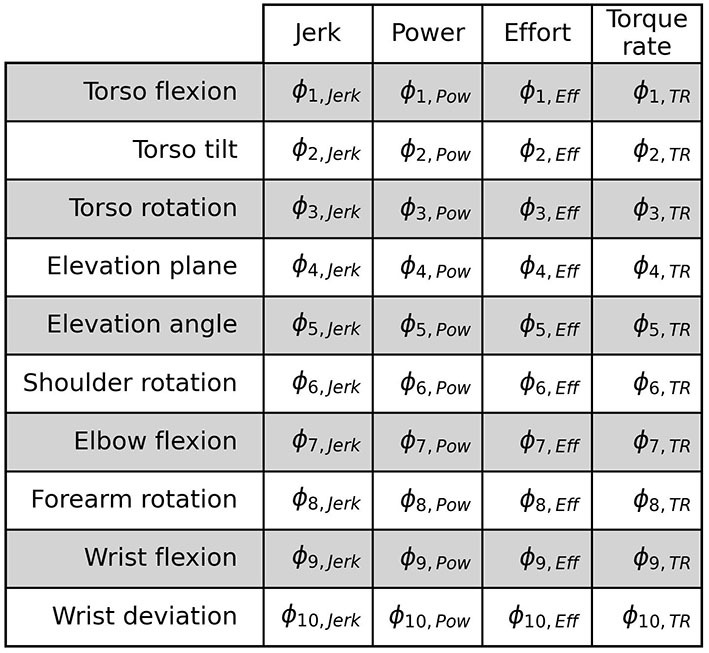
The CAE feature matrix. The rows depict the 10 DoFs and the columns depict the feature types. Each cell in the matrix corresponds to a feature.

Lastly, all features are normalized using min-max scaling between the values of 0 and 1 across the training dataset in order to remove any bias arising from numerically higher feature values (Singh and Singh, 2020). At test time, the feature values are scaled using the min-max values extracted from the training dataset. Since, we use leave-one-out cross-validation, feature normalization is done independently for each fold of evaluation. The resulting CAE feature matrix (see Figure 7) denoted by $\phi ={\left\{\phi_{d}\right\}}_{d=1}^{40}$ containing 40 scalar features forms one of the inputs to the classifier discussed in the Section 2.7.

### 2.6. Generation of the training dataset

For online classification, we generate the training dataset $D_{tr}$ by calculating the feature matrix for each time-step of each of the *N*_*M*_*tr*__ movement trajectories. This corresponds to the input feature vector **ϕ**_*t,m*_ comprising a single datapoint along with a ground-truth label. The latter is required by supervised learning methods. Since we have access to only sparse binary labels which we denote as *y*_*m*_ corresponding to the full *m*-th trajectory, we generate ground-truth labels for the intermediate steps of the trajectory by replicating the label corresponding to the full trajectory for all the frames, thus obtaining a label *y*_*t, m*_ for the *t*-th frame of the *m*-th motion trajectory. Assuming that the *m*-th trajectory has a length of *T*_*m*_, the full dataset D has a total of $\overset{N_{M_{tr}}}{\underset{m=1}{\sum }}T_{m}$ data-points. We can therefore succinctly represent our training dataset $D_{tr}$ as follows:


(1) $$\begin{array}{l} D_{tr}=\left\{\phi_{m,t_{m}},y_{m,t_{m}};\forall t_{m}\in \left\{1,\ldots ,T_{m}\right\},\forall m\in \left\{1,\ldots ,N_{M_{tr}}\right\}\right\} \end{array}$$

### 2.7. Classification algorithm

We train a linear classification model (Bishop and Nasrabadi, 2006) using supervised learning for discriminating between healthy and compensatory motions. More concretely, our goal is to learn a linear hyperplane ***w***·**ϕ**+*b* = 0, where the learnable parameters ***w*** and *b* are learnt such that the following conditions:


(2) $$\begin{array}{l} \begin{array}{c} w\cdot \phi +b<0\;\Rightarrow \;\text{Compensation}\\ w\cdot \phi +b\geq 0\;\Rightarrow \;\text{No Compensation} \end{array} \end{array}$$

are maximally satisfied over the training dataset $D_{tr}$.

Many different methods for learning the parameters ***w***, *b* have been described in the literature. We employ two popular approaches, namely linear Support Vector Machine (SVM) classification and Logistic Regression (LR) for learning these parameters. We describe these methods in the following subsections.

#### 2.7.1. Logistic regression

LR (Bishop and Nasrabadi, 2006) learns the parameters ***w***, *b* by minimizing the regularized cross-entropy loss on the training dataset.


(3) $$\begin{array}{l} L(w,b|D)=-\sum_{i=1}^{N_{D_{tr}}}\left[y_{i}\log\sigma (w\cdot \phi_{i}+b\right)\\ \;+(1-y_{i})\log(1-\sigma (w\cdot \phi_{i})+b)+\lambda ||w|{|}_{2}^{2}] \end{array}$$

where $\sigma \left(a\right)=\frac{1}{1+\exp\left(-a\right)}$ denotes the sigmoid function, ||·|| denotes the Euclidean norm, λ is the regularization constant, and *N*_*D*_*tr*__ demotes the size of the training dataset.

This loss has its basis in Maximum Likelihood Estimation (Bishop and Nasrabadi, 2006) which maximizes the probability *p*(**ϕ**) = σ(***w***·**ϕ**+*b*) of the input **ϕ** that belongs to the true class over the dataset $D_{tr}$. Once the classification model has been trained, the output of function *p*(.) can be interpreted as an indicator of the classification confidence. Thus, a probability value of 0.5 indicates that the classification model is uncertain regarding its prediction, while values closer to 0 or 1 indicate high model confidence.

#### 2.7.2. Support vector machine with a linear kernel

SVMs learn a linear classification hyperplane that can separate the positive and the negative class such that the margin for separating these two classes has the maximum distance. While SVM is a non-parametric method and typically remaps the input feature space to an infinite dimensional latent space when using complex kernels (Bishop and Nasrabadi, 2006), in this work, we use a linear kernel, that effectively translates to learning the model parameters ***w***, *b* by minimizing the Hinge Loss, that is given as follows:


(4) $$\begin{array}{l} L\left(w,b|D\right)=\overset{N_{D_{tr}}}{\underset{i=1}{\sum }}y_{i}\frac{1}{2}w^{T}w+\alpha \overset{N_{D_{tr}}}{\underset{i=1}{\sum }}\max\left(0,1-y_{i}\left(w\cdot \phi +b\right)\right) \end{array}$$

where α is a regularization parameter.

#### 2.7.3. Training hyper-parameters

Our proposed models with energy based features are both trained for 5, 000 iterations. The SVM model is trained with a squared-hinge loss and L2 penalty, while the LR model is trained with an LBFGS optimizer that minimizes the L2 -penalized cross-entropy loss described in Equation 4.

### 2.8. Identification of compensating joint

We propose to identify the compensating DoFs by exploiting a feature vector corresponding to each DoF independently. Given the parameters of the Ridge Regression model {***w***, *b*}, where $w={\left\{w_{d}\right\}}_{d=1}^{40}$ is the set of weights with a one-to-one correspondence with the elements of the feature matrix **ϕ**, we sort the list of 10 DoFs based on the corresponding weight-feature product given by:


(5) $$\begin{array}{l} \psi_{j}=w_{j,\text{Jerk}}\phi_{j,\text{Jerk}}+w_{j,\text{Pow}}\phi_{j,\text{Pow}}+w_{j,\text{Eff}}\phi_{j,\text{Eff}}+w_{j,\text{TR}}\phi_{j,\text{TR}} \end{array}$$

for the *j*-th DoF. If the class predicted by a trained model is “Compensated”, the DoF corresponding to the most negative weight-feature product contributes the most to that classification. This can be seen in Equation 2 which shows the linear combination of the weights and features per DoF determines the classification prediction by the model.

### 2.9. Online feature extraction and classification

For online classification, we must calculate the CAE features in an online manner from the input stream of joint kinematic and dynamic data. As noted in Section 2.5, creation of CAE features includes an aggregation process that presupposes the availability of segmented motion primitives. Therefore, similar to Section 2.3, we employ automated motion segmentation using zero-crossings of the end-effector velocity to extract goal-directed motion primitives from the trajectory. However, since for online classification, data is processed as a stream, and the full trajectory is revealed to us frame-by-frame, automatic segmentation is reapplied at regular intervals to update motion-primitive locations. The procedure for online segmentation is given as follows: (i) Maintain a history of *T*_*hist*_ previous observations of joint kinematics and dynamics, i.e ${\left\{q_{t},{\overset{\cdot }{q}}_{t},{\ddot{q}}_{t},\tau_{t}\right\}}_{t=1}^{T_{hist}}$ as well as marker positions ${\left\{m_{t}\right\}}_{t=1}^{T_{hist}}$. (ii) At regular intervals, recalculate the zero-crossing points from the marker history as well as update the observation history. (iii) Use the last observed zero-crossing point as the beginning of a motion primitive for calculating CAE features at time step *t* as described in Section 2.5.

### 2.10. Baseline models for comparison

We compare our approach of training the SVM and LR linear classifiers with energy-based features against 2 other baseline models. The first is an MLP with 2 hidden layers and 10 neurons in each layer. The second baseline is an LSTM with 2 hidden layers with 50 neurons each. Both architectures were employed in recent works for classification of compensatory motion (Zhi et al., 2017; Lin et al., 2021) and have served as baselines for other works that use deep learning architectures for analysing human motion data (Azmi and Sulaiman, 2017; Rustam et al., 2020; Wan et al., 2020; Ahad et al., 2021; Yao et al., 2021; Yu et al., 2021). LSTM, in particular has been considered to be suitable for modeling temporal processes, both in context of compensation detection as well as other domains. Since our objective is to demonstrate that exploiting prior bio-mechanical knowledge via energy-based features has competent performance to automatic feature extraction via deep neural networks, the input to our deep neural network baselines are joint-kinematic and dynamic trajectories [similar to Zhi et al. (2017)]. We use a sliding window of size *s* and an overlap of *s*−1 frames to create input vector at time-step *t* of size *s*×4 × *N*_*J*_. This is aligned with previous approaches which also look at a fixed-length window of data that slides along the trajectory, both for generating training datapoints and at test-time, for online classification (Zhi et al., 2017; Lin et al., 2021). Window length *s* was determined to be 100, corresponding to 1.0 seconds for MLP and 20, corresponding to 0.2 seconds for LSTM using grid-based hyper-parameter search. Further, the number of neurons in each layer of MLP and LSTM was also determined by using grid-based hyper-parameter search.

#### 2.10.1. Input normalization

Similar to the CAE features, before the input is accepted by either of the classification models, it is normalized using min-max scaling. Normalization is done independently for each fold of evaluation similar to the feature normalization for our proposed approach (Section 2.5.1).

### 2.11. Evaluation criteria

#### 2.11.1. Evaluation metrics and cross-validation

We evaluate our proposed approach against other approaches with three performance metrics - mean brier score, mean miss-classification rate and mean false discovery rate. Brier Score (BS) is used to measure the quality of uncertainty estimation of the model and can be formulated as $\text{BS}=\frac{1}{N}\overset{N}{\underset{i=1}{\sum }}y_{i}-p\left(\phi_{i}\right)$ where *N* is the size of the set over which BS is being calculated. A small value for BS indicates that the classification model is well calibrated. Miss-classification rate (MCR) measures the proportion of true class examples miss-classified as the other class. Note that MCR = 1 − recall, which is another popular metric for quantifying classification performance. Finally, false discovery rate (FDR) is calculated as the proportion of true class predictions that are incorrectly classified. We note that FDR = 1 − precision. Smaller values for each of these metrics (BS, MCR, FDR) is indicative of good classification performance.

All performance metrics are calculated for samples belonging to each class separately and take the mean. This is done to counterbalance the class-imbalance in the dataset which occurs since more acted compensation trajectories are collected than healthy trajectories.

Cross-validation for all evaluations is performed with leave-one-out approach (LOOCV), in order to ensure that we do not overfit to the test dataset (Cawley and Talbot, 2003).

#### 2.11.2. Significance testing

We additionally report the significance of classification performance among different methods. While it is common to use McNemar's test for this purpose, we cannot directly apply it on our test datapoints since they constitute different time-steps of the same trajectory and can be highly correlated, thus violating the independent samples assumption of the test. As a result, we instead use voting to aggregate the predictions at trajectory level where possible (model comparison and ablation study) and apply the Bonferroni-Holm correction to adjust the p-values whenever we conduct multiple comparisons. We deem the results to be statistically significant if *p* < 0.05. For each of the comparisons, we report statistics in the following format, χ^2^(degrees-of-freedom, *N* = number of samples) = value of statistic, *p* < 0.05 or *p* > 0.05.

Unfortunately, when assessing online classification performance, the test outputs always correspond to fractions of the same trajectory since the purpose of the experiment is to test the model performance on streaming data. This violates the independence assumption of McNemar's test. Consequently, significance testing with McNemar's test could not be conducted for this experiment.

## 3. Results

### 3.1. Model comparison

We compare the performance of our linear classifiers (SVM and LR) trained with energy-based features on the baseline deep learning models MLP and LSTM trained on raw observations (See Section 2.10 for a detailed description of the baseline models).

As indicated in Section 2.11.2, we use voting to aggregate the predictions at trajectory level to calculate the average performance metrics (Table 2). LR achieves the lowest mean mean BS (0.119) and MCR (0.137) amongst all the models. SVM has the lowest FDR (0.177). The highest mean BS (0.230) and FDR (0.281) is obtained by LSTM and the highest mean MCR (0.332) is obtained by MLP.

**Table 2:** Comparison of model performance using three metrics whose mean and standard deviation over all the test-folds generated using LOOCV are reported.

**Model**	**Brier score**	**Mis-classification rate**	**False discovery rate**
MLP	0.191 ± 0.05	0.332 ± 0.092	0.221 ± 0.094
LSTM	0.230 ± 0.109	0.270 ± 0.122	0.281 ± 0.169
SVM	0.119 ± 0.093	0.151 ± 0.124	**0.177** ±**0.103**
LR	**0.119** ±**0.060**	**0.137** ±**0.072**	0.194 ± 0.089

The bold entries indicate the smallest mean and corresponding standard deviation in the column.

Furthermore, we conduct McNemar's test to assess the significance of model performances. LR significantly outperforms MLP, $\chi^{2}\left(1,N_{k}=600\right)=8.51,p<0.05$, as well as LSTM, $\chi^{2}\left(1,N_{k}=600\right)=5.78,p<0.05$. No significant differences were found between the classification performance of SVM and LR, χ^2^(1, *N* = 600) = 5.54, *p*>0.05. SVM significantly outperforms MLP, χ^2^(1, *N* = 600) = 23.36, *p* < 0.05, and LSTM, χ^2^(1, *N* = 600) = 17.69, *p* < 0.05. Finally, the classification performance of MLP was not found to be significantly different from that of LSTM, χ^2^(1, *N* = 600) = 0.653, *p* > 0.05.

**Figure 8:**
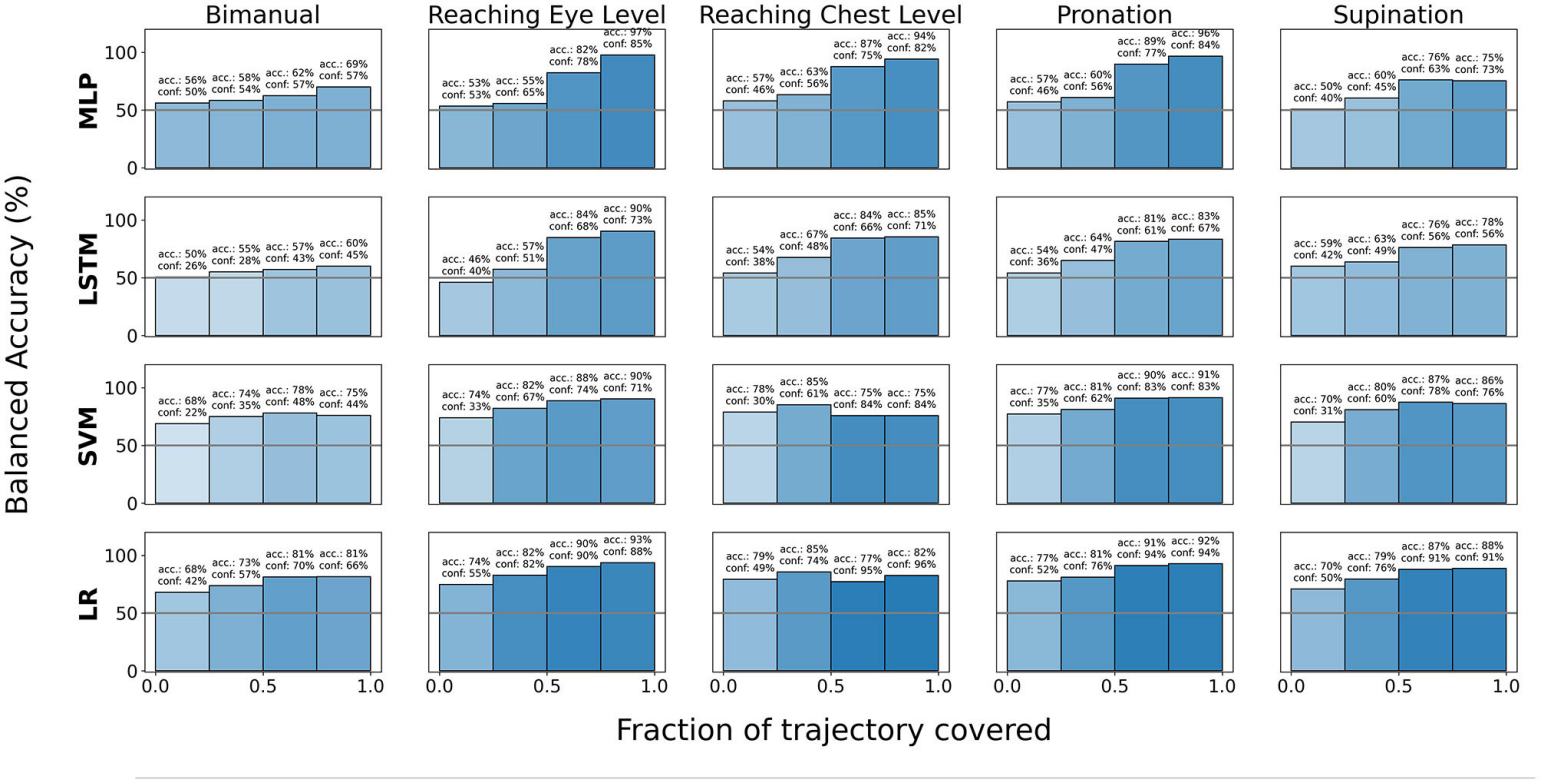
Balanced accuracy computed via LOOCV for various models as one progresses along the trajectory horizon for various pre-segmented motion primitives. The color gradient of the bars indicate the average model confidence at the corresponding time-step of the trajectory.

We additionally report averaged balanced accuracy (i.e the mean of “Healthy” and “Compensation” classification accuracies) separately for each temporal inter-quartile-range of the trajectory where the temporal-quartiles describe the fraction of the trajectory covered. These results are categorized by type of motion primitive and illustrated in Figure 8. Balance accuracy (BA) for each bin *B* with *N*_*B*_*heal*__ healthy datapoints and *N*_*B*_*comp*__ compensatory datapoints is calculated as:


$$\begin{array}{l} \text{BA}=\frac{1}{2}\left(\frac{\overset{N_{B_{heal}}}{\underset{i=1}{\sum }}𝟙|\phi_{i}^{B_{heal}}\text{classified as “Healthy”}}{N_{B_{heal}}}+\frac{\overset{N_{B_{comp}}}{\underset{i=1}{\sum }}𝟙|\phi_{i}^{B_{comp}}\text{classified as “Compensatory”}}{N_{B_{comp}}}\right) \end{array}$$

#### 3.1.1. Online classification results

We validate our proposed method for online feature extraction and classification in this section. Our test data for each fold from LOOCV comprises of the continuous trajectories of repeated motions (healthy or compensatory) collected from the corresponding “Left Out” participant before the data-processing step of automatic segmentation. We process the whole continous trajectory frame-by-frame at the rate of 100 Hz for both our energy-based linear classifiers, as well as the baseline methods (MLP and LSTM). Thus at each time-step *t*, we have access to only the first *t* frames. We use the method proposed in Section 2.9 for automatic segmentation and online extraction of the CAE features. We contrast the method proposed for online feature extraction in Section 2.9 with a simple method that assumes that all the non-segmentable motion primitive have a length of 1 second and are contiguous. In the case of the deep learning baselines MLP and LSTM, we use a First In First Out (FIFO) buffer for obtaining the windowed input. These buffers have the same length as the sliding windows used for training the models. Thus, *s* = 100 (corresponding to 1.0 seconds) for MLP and *s* = 20 (corresponding to 0.2 seconds) for LSTM.

Thus, for all the approaches, we obtain a predicted class for each time-step of the trajectory. However, during the calculation of evaluation metrics, we exclude test datapoints that correspond to the portion of the trajectory where the participant is returning to the start position after executing the motion primitive since participants are not explicitly asked to follow any protocol during this portion of the motion. We report our results in Table 3. Our experiment shows that when automatic segmentation employed, our method (LR (auto-seg) achieves the lowest mean BS (0.134), MCR (0.168), and FDR (0.228). The highest mean BS (0.217), MCR (0.328), and FDR (0.342) is achieved by LSTM.

**Table 3:** Comparative performance of different models on online classification tasks.

	**Brier score**	**Misclassification rate**	**False discovery rate**
MLP	0.199 ± 0.047	0.298 ± 0.066	0.299 ± 0.065
LSTM	0.217 ± 0.059	0.328 ± 0.080	0.342 ± 0.068
SVM (fixed-seg)	0.188 ± 0.036	0.233 ± 0.072	0.313 ± 0.036
LR (fixed-seg)	0.169 ± 0.056	0.223 ± 0.075	0.302 ± 0.039
SVM (auto-seg)	0.154 ± 0.05	0.171 ± 0.085	0.237 ± 0.048
LR (auto-seg)	**0.134** ±**0.075**	**0.168** ±**0.085**	**0.228** ±**0.051**

The mean and standard deviation of three evaluation metrics over the folds of LOOCV are reported here. The bold entries indicate the smallest mean and corresponding standard deviation in the column.

#### 3.1.2. Uncertainty estimation comparison

**Figure 9:**
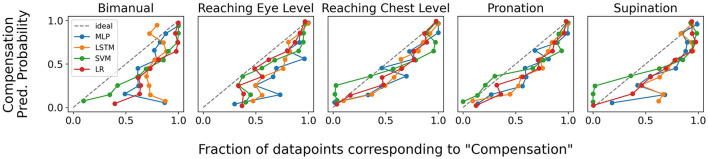
Calibration plots demonstrating the quality of uncertainty quantification by various models for the 5 different movement primitives. The closer the plot is to the ideal classifier, the better the uncertainty estimation quality of the model.

In addition to model accuracy, the quality of uncertainty estimation is also an important factor for measuring the performance of classification models. As discussed previously, BS is one approach toward quantifying model uncertainty estimation performance and has been reported for various models in Tables 2, 3. We additionally provide qualitative results in the Figure 9 in the form of calibration plots. We note that while none of the models are perfectly calibrated, SVM and LR, both of which use CAE features, are the closest to the ideal classifier.

### 3.2. Compensating joint identification

**Figure 10:**
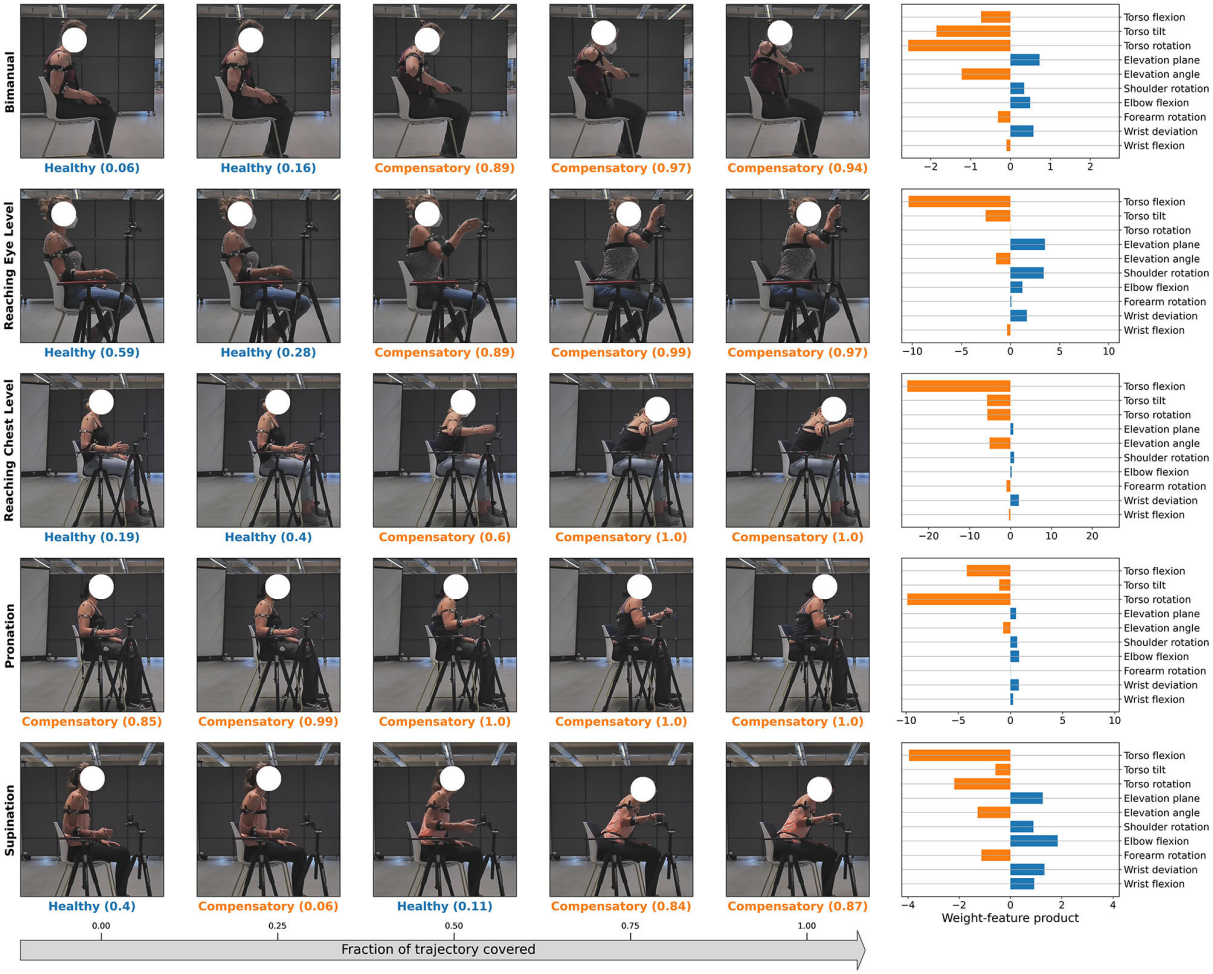
Examples of compensating joint identification using the LR classification model for various tasks with acted compensations are shown. The model's classification prediction is indicated along with the model confidence score. The joints that contribute to the classification “Compensatory” in last frame of the sequence are shown in the last column along with their corresponding weight-feature product. The more negative the value for this product is, the greater the possibility that the corresponding joint is being used for compensation. Positive values of this product indicate that the corresponding joint is used in typical movements.

In this section, we show qualitative results for the identification of compensating joints in Figure 10. All the sample trajectories shown in the Figure belong to the “Compensated” class and were randomly sampled from the same class. However, note that despite this, some of the early frames of the motion are classified as “Healthy”. Since all our test trajectories begin with a healthy pose, this result is in accordance with our expectations. We note that the weight-feature product also potentially provides an interpretable way of identifying the compensating joints as well as the relative degree of compensation. The more negative the weight-feature product corresponding to a DoF, the more likely it is that the DoF is contributing to a compensation classification.

### 3.3. Ablation study of feature aggregation

Lastly, we conduct an ablation study of the feature aggregation step (Section 2.5) for LR models to evaluate the benefit of aggregating the trajectory features until current time step *t* with an averaging function. We compare our proposed aggregation mechanism against other feature aggregation mechanisms, including no aggregation, where no feature aggregation is performed, and windowed aggregation, where instead of calculating the cumulative average, the final feature at each time step *t* is calculated as the average of the feature values over a window of size *s*∈{20, 100} centered at time step *t*, corresponding to 0.2 and 1.0 seconds respectively.

Similar to Section 3.1, we use voting to aggregate the predictions at trajectory level to calculate the average performance metrics (Table 4). Using CAE features yields the lowest mean MCR (0.137) and lowest mean FDR (0.194). The lowest mean BS (0.110) is achieved by using features averaged over a 1 second long window i.e, “Windowed Avg Feature (1s)”. Models that donot use any feature aggregation yield the highest mean BS (0.168), highest mean MCR (0.229), and highest mean FDR (0.286).

**Table 4:** Results from the ablation study for determining the best aggregation over features.

**Feature aggregation mode**	**Brier score**	**Mis-classification rate**	**False discovery rate**
Without Feature Aggregation	0.168 ± 0.034	0.229 ± 0.059	0.286 ± 0.03
Windowed Avg Feature (0.2s)	0.140 ± 0.039	0.188 ± 0.048	0.260 ± 0.038
Windowed Avg Feature (1s)	**0.110** ±**0.051**	0.138 ± 0.079	0.202 ± 0.091
Cumulative Avg Feature	0.119 ± 0.060	**0.137** ±**0.072**	**0.194** ±**0.089**

The mean and standard deviation of all three metric values are calculated over LOOCV folds. The bold entries indicate the smallest mean and corresponding standard deviation in the column.

Statistical testing with McNemar's test reveals that models trained with CAE features significantly outperform models that do not use feature aggregation, i.e., “Without Feature Aggregation”, χ^2^(1, *N* = 600) = 65.29, *p* < 0.05, as well as models using features averaged over a 0.2 seconds long window i.e, “Windowed Avg Feature (0.2s)”, χ^2^(1, *N* = 600) = 65.29, *p* < 0.05. However, the McNemar's test is unable to show a significant difference between the classification performances of models using CAE features and models with features averaged over a 1 second long window i.e, “Windowed Avg Feature (1s)”, χ^2^(1, *N* = 600) = 0.02, *p*>0.05. Among other comparisons, models with “Windowed Avg Feature (1.0s)” significantly outperform models trained on “Windowed Avg Feature (0.2s)”, χ^2^(1, *N* = 600) = 34.68, *p* < 0.05, as well models trained without feature aggregation, χ^2^(1, *N* = 600) = 69.89, *p* < 0.05. Finally, models with “Windowed Avg Feature (0.2s)” significantly outperform models trained without feature aggregation, χ^2^(1, *N* = 600) = 30.04, *p* < 0.05.

## 4. Discussion

The aim of this work was to validate a novel approach for automatically detecting compensation strategies with an analytical capability from the kinematic and dynamic trajectory of a motion. For this purpose, we trained a linear classifier on energy-based features. In order to identify the individual joints contributing to compensation, these features were calculated independently for each observable DoF in the distinct segments of the upper body. Temporal information was encoded by aggregating the energy-based features along the input trajectory using cumulative averaging. Two typical methods for learning the linear classifier, namely SVM and LR were investigated.

Our proposed method was validated on a dataset of 5 motion primitives collected from 6 physiotherapists with healthy movements as well as acted compensations. This allowed us to collect a larger variety of compensatory behavior as observed by experienced physiotherapists in contrast to simulating just three types of compensatory behaviors (Zhi et al., 2017). Furthermore, previous approaches (Zhi et al., 2017; Cai et al., 2019; Ma et al., 2019) only collected a dataset for uni-manual reaching tasks. In contrast, we record the motions for a bi-manual task (lifting a tray), as well as tasks requiring pronation and supination.

We compared our method against two deep learning baselines MLP and LSTM (Section 2.10) that can perform automatic feature extraction, and have been used in previous approaches for compensation detection (Zhi et al., 2017; Khoramdel et al., 2021; Lin et al., 2021).

Comparison of evaluation metrics (Table 2) and statistical testing with McNemar's test (Section 3.1) showed our methods (LR and SVM) significantly outperformed deep learning baselines, namely MLP and LSTM. In contrast, in Zhi et al. (2017), classification performance of LSTM was noted to be similar for healthy participants and better for stroke patients compared to SVM (without energy-based features). Our results thus indicate that including prior biomechanical information in the form of energy-based features can allow for superior classification performance compared to deep learning approaches, even when simple linear classification methods are used. These results are very promising since both baseline models that exploit deep learning have a larger number of trainable parameters by design and are therefore susceptible to overfitting to the training data (Bishop and Nasrabadi, 2006).

We additionally validated our proposed online classification mechanism. Table 3 showed that our proposed methods, LR and SVM models yielded lower values of mean BS, MCR and FDR compared to LSTM. Furthermore, these models yielded lower values of mean BS and MCR; and lower or similar values of FDR compared to MLP. In practice, lower values of these metrics corresponds to better classification performance. This indicates that, equipped with energy-based features, models with less trainable parameters such as LR and SVM can perform online classification for compensation detection as well as deep learning-based models that have considerably more parameters. Since training time scales with the size of the model, and can consequently impact online classification computation time in cases where model retraining is needed (such as continual learning Hadsell et al., 2020), this is a promising result.

Within the context of online classification, we studied two different methods for online segmentation of motion primitives for calculating CAE features. Our preferred method, automated motion-primitive segmentation using zero-crossing of velocities (Section 2.3) achieved lower values of BC, MCR, and FDR compared to a method that assumes the full motion to be composed of contiguous segments, each with a fixed length of one second. However, the method that we use for automated motion-primitive segmentation can be limited in its applicability since it strongly depends on how cleanly the movement data is separable into primitives by instances of zero velocity, which is not always possible in real scenarios. In a real-world setting, natural human movements may contain hesitation and noise, and therefore multiple points of zero-crossing velocities can be observed, despite not corresponding to the actual beginning or end of a motion primitive. In such situations, assuming a fixed segmentation-length for the calculation of CAE features can still give acceptable results. Many additional methods exist for primitive motion segmentation in literature. For instance, Barbič et al. (2004) uses probabilistic principal component analysis to track changes in the motion distribution and find segmentation points; Beaudoin et al. (2008) uses k-Nearest Neighbors to cluster individual motion frames, associate different clusters with a unique symbol and subsequently partition the complete movement based on identification of different cluster sub-sequences; Kulić et al. (2012) uses clustering and hidden markov models (HMM) for online segmentation; and Zhou et al. (2022) uses transfer learning to learn a segmentation model from related motion data that already has segmentation labels. We leave the investigation and validation of these methods and their robustness to real-world noisy data for compensation detection to future works.

Our proposed approach for compensation joint identification (Section 2.8 and Figure 10), potentially provides a straightforward and interpretable way of identifying compensating joints, as well as the degree of compensation being performed without having to rely on detailed-annotations of compensation locations as opposed to previous approaches (Zhi et al., 2017; Cai et al., 2019; Ma et al., 2019; Kashi et al., 2020). Visual verification of the randomly sampled trajectories in the figure indicates the method can successfully identify the compensating joints. However, this result is only a qualitative observation since the ground truth labels for loci of true compensation were not a part of the dataset annotation. Full validation of this technique via collection of annotations for a validation set and comparison of the prediction results with the same is left to future work.

We additionally investigated the impact of trajectory progress toward the final kinematic pose on classification accuracy and model confidence. Our analysis in Figure 10 indicates that model confidence tends to increase as the time step *t* of the trajectory progresses. This is more clearly observed in Figure 8 at a macro level, where both the model confidence and accuracy tends to increase as the trajectory progresses for all the model architectures we studied. However, we also noted that for most cases, model accuracy reached higher magnitudes earlier for linear classification models (LR and SVM) compared to the deep learning baselines (MLP and LSTM). We believe that this is owed to including prior biomechanical information in the linear classifiers inputs in form of energy-based features, which, as we have already shown in Section 3.1, outperform baseline deep learning methods.

Regarding model performance across various tasks, the analysis presented in Figure 8 indicates that classification accuracy is relatively lower for bimanual tray lifting compared to other tasks. This suggests that the difference between healthy and compensatory behavior in terms of joint kinematics and dynamics during bimanual motions is inherently dissimilar to that during uni-manual motions. If that is the case, ensemble-based models, where each model is trained to identify compensations for individual task types can be used. However, additional data and analysis is required for a thorough investigation of this dissimilarity.

We also studied model confidence more closely in calibration plots shown in Figure 9. Both a qualitative review of these results along with the BS reported in Tables 2, 3 show that LR and SVC models are better calibrated than the deep learning-based MLP and LSTM models. This implies that the uncertainty values estimated by our linear classification models is more reliable than those predicted by the deep learning baselines. This result is consistent with previous literature which link overfitting (more commonly observed in deep neural networks) to uncertainty calibration (Guo et al., 2017; Mukhoti et al., 2020).

Lastly, we conducted an ablation study to determine the best mode of feature aggregation. We found that models trained with CAE features significantly outperform models trained without feature aggregation or with features averaged over a sliding window of size 0.2 seconds. We speculate that the success of CAE features can be attributed cumulative averaging of the features over the seen trajectory, which helps encode the trajectory history in the feature and leads to better classification. We note however, that averaging features over a larger window (with length 1 second) does not lead to a significantly different classification performance than using CAE features. A possible reason could be that a window of length 1 second is able to capture enough temporal information for accurate classification. However, the window size is still an additional hyper-parameter to be optimized when the mode of feature aggregation is chosen to be averaging over a fixed length window. In contrast, using CAE features does not require any such hyper-parameter optimization.

While this study proposes a novel method for compensation detection and compensating joint identification from sparse labels, which, to our knowledge has not been explored before in literature, it also has a few limitations that must be considered during application of this approach, and can be improved upon in future extensions of this work.

Even though all of our participants, being experienced physiotherapists, are familiar with common compensation strategies and drew from experience while simulating compensated motions, variability of compensation strategies in patients can be higher since multiple factors, such the stroke severity (Levin et al., 2016) and fatigue (Zhi et al., 2017) and their combinations can affect the type and degree of compensation employed in different ways. Compensations arising from milder impairments may be only slightly discernible and consequently harder to classify (Zhi et al., 2017). Future work will therefore explore the robustness of the methods established in this approach on motion data collected from patients.

Furthermore, this study comprised of analysing 5 motion primitives, most daily living tasks are much more complex and are composed of different primitive motions. We will therefore verify the robustness of this approach on a dataset of more complex motions in the future.

Additionally, this work strictly imposes the condition of calculating features that are independent for each observable DoF in order to infer compensating joints. However, low coordination between 2 or more DoFs has also been noted to be an indicator of compensation (de Los Reyes-Guzmán et al., 2017). Thus, incorporating features measuring joint-coordination such as movement correlation (de Los Reyes-Guzmán et al., 2017) can be a promising line of investigation for subsequent studies.

Finally, the problem of compensation detection from kinematic data is closely related to other applications for human motion classification such as human activity recognition Vrigkas et al. (2015) and gait analysis Yao et al. (2021). Many of these works therefore deal with similar challenges, such as processing temporal data, online classification and multilabel classification. Consequently, future works can also investigate novel solutions from these works for compensation detection. For instance, Chamroukhi et al. (2013) proposed a method for automated motion segmentation for human activity recognition using expectation-maximization and HMM which can also be investigated for online classification using our approach; and Yao et al. (2021) combined different temporal features such as time-domain, frequency-domain and wavelet-domain based features for gait analysis which can also be used to extend the CAE feature set proposed in this work for compensation detection.

## 5. Conclusion

Reliable identification of compensatory strategies in post-stroke patients is crucial for the long-term recovery of the patient. Current methods rely on densely annotated training datasets that can be cumbersome to acquire. To mitigate this, we propose to train a linear classifier with energy-based features that can automatically classify disjointed motion primitives as healthy or compensatory in addition to identifying the compensating joints from sparsely labeled training data. We acquired a dataset of 5 motion primitives including bimanual lifting and uni-manual reaching tasks executed by 5 healthy physiotherapists multiple times, both with and without simulated compensations. The methods proposed in this were validated on the aforementioned dataset using leave-one-out cross validation and outperformed deep learning-based methods that are parameter heavy and are more difficult to train. Future studies will verify the methods proposed in this work on data collected from actual stroke patients.

## Data availability statement

The raw data supporting the conclusions of this article will be made available by the authors, without undue reservation.

## Ethics statement

The studies involving human participants were reviewed and approved by Ethics Committee of the Medical Faculty of the Technical University of Munich. The patients/participants provided their written informed consent to participate in this study.

## Author contributions

Conceptualization: ND, SE, CK, and SH. Software and data processing: ND and SE. Validation, writing—original draft preparation, and visualization: ND. Data acquisition: ND and SP. Writing—review and editing: ND, SE, SP, CK, and SH. Supervision: SE, CK, and SH. Funding acquisition: CK and SH. All authors have read and agreed to the published version of the manuscript.
